# *Lactobacillus plantarum* Lp2 improved LPS-induced liver injury through the TLR-4/MAPK/NFκB and Nrf2-HO-1/CYP2E1 pathways in mice

**DOI:** 10.29219/fnr.v66.5459

**Published:** 2022-07-05

**Authors:** Yiying Chen, Wuyang Guan, Nan Zhang, Yu Wang, Yuan Tian, Haiyue Sun, Xia Li, Yuhua Wang, Jingsheng Liu

**Affiliations:** 1 College of Food Science and Engineering, Jilin Agricultural University, Changchun, China; 2 Jilin Province Innovation Center for Food Biological Manufacture, Jilin Agricultural University, Changchun, China; 3 National Processing Laboratory for Soybean Industry and Technology, Changchun, China; 4 National Engineering Laboratory for Wheat and Corn Deep Processing, Changchun, China

**Keywords:** Lactobacillus plantarum Lp2, liver injury, oxidative stress, inflammation response, TLR-4/MAPK/NFκB, Nrf2-HO-1/CYP2E1

## Abstract

**Background:**

Inflammatory liver diseases present a significant public health problem. Probiotics are a kind of living microorganisms, which can improve the balance of host intestinal flora, promote the proliferation of intestinal beneficial bacteria, inhibit the growth of harmful bacteria, improve immunity, reduce blood lipids and so on. Probiotics in fermented foods have attracted considerable attention lately as treatment options for liver injury.

**Objective:**

The aim of this study was selected probiotic strain with well probiotic properties from naturally fermented foods and investigated the underlying mechanisms of screened probiotic strain on lipopolysaccharide (LPS)-induced liver injury, which provided the theoretical foundation for the development of probiotics functional food.

**Design:**

The probiotic characteristics of *Lactobacillus plantarum* Lp2 isolated from Chinese traditional fermented food were evaluated. Male KM mice were randomly assigned into three groups: normal chow (Control), LPS and LPS with *L. plantarum* Lp2. *L. plantarum* Lp2 were orally administered for 4 weeks before exposure to LPS. The liver injury of LPS-induced mice was observed through the evaluation of biochemical indexes, protein expression level and liver histopathology.

**Results and discussions:**

After treatment for 4 weeks, *L. plantarum* Lp2 administration significantly reduced the LPS-induced liver coefficient and the levels of serum or liver aspartate transaminase (AST), alanine aminotransferase (ALT), tumor necrosis factor α (TNF-α), interleukin-6 (IL-6) and LPS, as well as decreasing the histological alterations and protein compared with the LPS group. Western-blotting results showed that *L. plantarum* Lp2 activated the signal pathway of TLR4/MAPK/NFκB/NRF2-HO-1/CYP2E1/Caspase-3 and regulated the expression of related proteins.

**Conclusions:**

In summary, *L. plantarum* Lp2 suppressed the LPS-induced activation of inflammatory pathways, oxidative injury and apoptosis has the potential to be used to improve liver injury.

## Popular scientific summary

- *L. plantarum* Lp2 with great probiotic characteristics screened from Chinese traditional food and identified.
- *L. plantarum* Lp2 decreased the secretion of inflammatory cytokines (TNF-α and IL-6).
- *L. plantarum* Lp2 alleviates LPS-induced liver injury via reducing oxidative stress.
- *L. plantarum* Lp2 mitigates LPS-induced liver injury via activating Nrf2-HO-1/CYP2E1 pathway and inhibiting TLR-4/MAPK/NFκB pathway.

Many factors cause liver injury including fatty liver, inflammation, fibrosis, cirrhosis and even liver cancer. Previous studies showed that alcohol and high-fat diet can induce the increase of blood lipopolysaccharides (LPS) that contribute to liver injury (1–3). Normally LPS derived from the cell wall of Gram-negative bacteria almost penetrate the gut epithelium for the intact intestinal barrier function; however, LPS leakiness may be increased in blood and accumulated in liver under certain pathological conditions, such as acute alcohol abuse (4). The liver is the most important metabolic organ and plays a key role in the host defense response because of its ability to remove pathogenic microorganisms and toxins (5). And it is also the main victim of these attacks, leading to the activation of host immune cells, inciting inflammation (6). The undesirable inflammatory reaction not only damages the liver function defense ability but also causes a large number of hepatocyte necrosis, leading to liver injury and eventually triggering liver failure (7, 8). The overexpression of pro-inflammatory cytokines and the production of reactive oxygen species (ROS) can lead to liver injury (9).

Lactic acid bacteria (LAB) are industrially important microorganisms worldwide for the fermentation of foods. And LAB as probiotics have a variety of biological effects, such as reducing the incidence of obesity-related metabolic diseases (2), improving body immunity (10), preventing cancer (11), playing the role of anti-inflammation (12) and antioxidation (13). Probiotics are live microorganisms with some very important characteristics, including acid tolerance, bile salt tolerance, adhesion to intestinal epithelial cells and antibacterial activity (14). FAO and WHO have established some basic criteria for screening probiotic strains, including checking tolerance to gastrointestinal transport, generating activity against pathogenic microorganisms, adhesion to human intestinal mucosa and required immunity regulate activity (15, 16). *L. plantarum* was used to alleviate high cholesterol, constipation, inflammatory bowel disease, vaginitis, allergies, etc. and to improve the flavor and nutrition of fermented foods, including Korean kimchi, European cheese, Chinese tofu, Italian sausage and so on, account for a large part of our daily diet in different diet cultures around the world. Previous studies have shown that both soymilk (17) and ginseng (18) fermented with probiotics can alleviate liver injury and fatty liver disease (19). Recent studies have shown that specific *L. plantarum* used in the fermented foods can exert probiotic properties in a strain-dependent manner (3). *L. plantarum* A41 screened by Lee and Kim inhibited the expression of inflammatory mediators stimulated by LPS and improved immune-related bone health (20). However, the mechanism of *L. plantarum* on LPS-induced liver injury remains obscure.

In this study, the probiotic characteristics of *L. plantarum* Lp2 screened from pickled cabbage were measured by in vitro experiments, cell experiments and animal model tests. The protective effects and the molecular mechanisms of *L. plantarum* Lp2 on liver injury in LPS-induced mice were analyzed.

## Materials and methods

### Materials

The *L. plantarum* Lp2 strain was isolated from pickled cabbage purchased from the agricultural market (Changchun, China). Man-Rogosa-Sharpe (MRS) agar medium and LB agar medium were purchased from BD Biosciences-Advanced Bioprocessing (Franklin Lake, New Jersey). All chemicals needed for the oro-gastrointestinal tract assay (lysozyme, pepsin, pancreatin, bile salts), Dulbecco’s modified Eagle medium (DMEM), RPMI medium, fetal bovine serum (FBS), phosphate buffered saline (PBS), l-glutamine, penicillin, streptomycin, and LPS were obtained from Sigma-Aldrich Co. (St. Louis, MO). RIPA cell lysis buffer was obtained from Solarbio Science & Technology Co. Ltd (Beijing, Chain). Ezup column bacteria genomic DNA purification kit was obtained from Sangon Biotech Shanghai Co. Ltd (Shanghai, China). The commercial assay kits of aspartate aminotransferase (AST), alanine aminotransferase (ALT) and dye kits hematoxylin and eosin (H&E) were purchased from Nanjing Jiancheng Bioengineering Institute (Nanjing, China). BCA protein assay kit was obtained from Thermo Scientific (Shanghai, China). The enzyme-linked immunosorbent assay (ELISA) kit of mouse LPS, tumor necrosis factor-alpha (TNF-α) and interleukin (IL)-6 was obtained by Solarbio Science & Technology Co., Ltd (Beijing, China). Antibodies directed against nuclear factor-like 2 (Nrf2), heme oxygenase 1 (HO-1), cytochrome P450 2E1 (CYP2E1), caspase-3 and Toll-like receptor 4 (TLR4) were purchased from Abcam (Cambridge, MA). Antibodies directed against IκBα, NFκB, phospho-IκBα, phospho-NFκB, p38 MAPK, ERK, JNK, c-Jun, phosphor-p38 MAPK, phosphor-ERK, phosphor-JNK and phosphor-c-Jun were purchased from Cell Signaling Technology (Danvers, MA). The secondary antibodies for western blot were received from Cell Signaling Technology (Danvers, MA). All of the other reagents were of analytical grade.

### Bacterial strains, cells and culture conditions

#### Isolation and strain culture

The strains, isolated from a commercially available pickled cabbage, were cultured for 24 h at 37°C under anaerobic conditions in MRS broth. The viability of the strain was determined by plate counting. These were counted by diluting and streaking on MRS agar plates, followed by the overnight culture at 37°C. Screening strains were cultured in a liquid MRS medium for 18 h until they reached a bacterial density of 1 × 10^9^ CFU/mL. Based on the observation of colony morphology and microscopic examination, the pure strain was obtained by repeated steps of separation and purification. The bacteria precipitate and supernatant (Lp2s) were separated by centrifuged at 4°C, 5,000 g for 10 min, and then the harvest cells were washed twice and adjusted to 1 × 10^9^ CFU/mL with sterile saline.

#### Strain identification

The pure strain was identified via 16S rDNA sequencing. DNA was extracted using the Ezup column of a bacterial genomic DNA extraction kit. After PCR amplification and purification, the samples were sequenced by Shanghai Biotechnology Engineering Co. Ltd. The 16S rDNA sequencing results were input into GenBank for homology comparison, and Kimura’s two-parameter distance correction model was used to construct phylogenetic trees via MEGA 7.0 software.

#### Caco-2 cell culture and treatment

The human colon cell line Caco-2 obtained from Shanghai Fuheng Biotechnology Co., Ltd. Caco-2 cells were grown in DMEM supplemented with 10% (v/v) heat-inactivated fetal bovine serum, 2 mM l-glutamine, 50 U/mL penicillin and 50 μg/mL streptomycin, at 37°C with 5% CO_2_. Caco-2 cells were seeded in 96-well cell culture plates at 1.25 × 10^5^ cells per well and cultivated for 3 weeks in order to obtain steady monolayers. The medium was changed every 2 days. Cell culture method was performed as previously described (14).

### In vitro probiotic properties assays

#### Oro-gastro-intestinal transit assay

First, 0.1 g of the bacteria precipitate was added to 10 mL simulated gastric juice (SGJ) and incubated at 37°C for 0, 2 and 4 h to determine total survival. Then, simulated intestinal juice (SIJ) was used instead of SGJ and incubated at 37°C for 0, 2 and 4 h to determine the total survival. The preparation of simulated gastrointestinal juice refers to the method of Zhang et al. (21).

#### Antibacterial activity test

The antibacterial activity of *L. plantarum* Lp2 supernatant against three human pathogens, namely *E. coli*, *S. typhimurium* and *S. aureus* was determined by agar well diffusion method. Lp2s were filtered by 0.45-μm filter. Among them, a part of the supernatant was adjusted to pH 7.0 as a control. Pathogen strains were spread onto the surface of LB agar. Wells of diameter 5 mm were bored, and Lp2s were loaded into each well. The zones of inhibition were measured after incubation at 37°C for 24 h.

#### Caco-2 cell adhesion test

The complete growth medium was replaced with absolute DMEM for 24 h before the adhesion assay. *L. plantarum* Lp2 was resuspended in DMEM and incubated with Caco-2 cells (1 mL Lp2 per well) at 37°C and 5% CO_2_ for 2 h. The wells were washed with PBS; and then Caco-2 cells and adherent bacteria were separated by adding trypsin (0.1 mL per well) and resuspended in PBS. The number of bacteria attached to the cells was determined by plating serial dilutions on MRS agar. The adhesion rate was calculated by comparing with the Colony Forming Unit (CFU) values of the washed wells (cell-bound bacteria only) and the control unwashed wells (unbound and bound bacteria).

### Animal treatment

Male KM mice (6 weeks, 18–22 g; Liaoning Changsheng biotechnology, Ben Xi, China) were housed in the Central Animal Facility (CAF) at the Jilin Agricultural University at 22–25°C, 55 ± 15% humidity, and 12 h light–dark cycle with unrestricted access to standard mouse chow and water and housed. The mice were randomized into three groups (*n* = 10 for each group): control group, LPS group, and Lp2 + LPS group (Lp2 group); *L. plantarum* Lp2 was mixed with drinking water at a ratio ensuring one mouse consumed 1 mL supernatant a day, and the body weight of the mice was recorded every week. All mice were fasted for 12 h after 4 weeks of dieting. LPS group and Lp2 group were followed by single intraperitoneal (i. p.) injection of 2.5 mg/kg LPS. Plasma and tissue samples were collected for assays at 6.0 h after the last injection. All mice were treated according to protocols reviewed and approved by the Institutional Animal Care and Use Committee of Jilin Agricultural University (SCXK-2016-0006).

### Determination of organ coefficient

The weights of liver, thymus and spleen were recorded. The organ coefficient was calculated according to the following formula:

Organ coefficient(mg/g)=organ weight (mg) / body weight (g) .

### Biochemical assays for the serum and liver tissues

The contents of ALT and AST in serum were quantified using commercial assay kits. The contents of LPS, TNF-α and IL-6 in serum and hepatic were determined using ELISA assay kits. The absorbance was measured at 450 nm in an ELISA reader (TECAN, Mannedorf, Switzerland).

### Histological analysis

The histopathological characters were used for evaluating liver histological damage, including hepatocyte necrosis, inflammatory cell infiltration and intrahepatic hemorrhage. For the liver histopathological assessment, fresh tissues were kept in 10% neutral buffered formalin solution. The tissues were embedded in paraffin and cut into 5-μm thick sections. After H&E staining, the histopathological examination was subsequently observed with sections under a light microscope (Leica, Heidelberg, Germany).

### Western blot analysis

The liver tissues were placed in RIPA cell lysis buffer and homogenized. The liver lysate was centrifuged at 12,000 rpm for 10 min at 4 °C, after that it was stayed on ice for 30 min. The supernatant was removed, and the total protein content was measured by using the BCA protein assay kit. Equivalent amounts of protein extracts (40 μg/lane) were loaded onto 8%, 10% or 12% SDS-PAGE; proteins were transferred to PVDF membranes, which the membranes were blocked with 5% skim milk in Tris-buffered saline-T (0.1% Tween-20 in TBS) more than 1 h at room temperature. After transferring, the membranes were incubated with specific primary antibodies at 4°C overnight. Antibodies against TLR4, JNK, p-JNK, ERK1/2, p-(ERK1/2), p38, p-p38, c-Jun, p-c-Jun, NFκB (p65), p-p65, IκBα, p-IκBα, Nrf2, HO-1, CYP2E1 and Caspase-3 visualized by enhanced chemiluminescent by using horseradish peroxidase-conjugated antibody. The protein bands were visualized by an image scanner (iBright CL1000, Thermo Fisher Scientific, USA). Protein levels were normalized to β-actin. The density of protein bands was quantified using Image J software (Materialize NV, Leuven, Belgium).

### Statistical analysis

Both the biological experiments and analytical tests were conducted at least three times. The data were expressed as the mean ± SEM. All data were analyzed by analysis of variance (one-way ANOVA) that was carried out using GraphPad Prism 7.0 (Prism 7.0 Software package, La Jolla, USA). Differences were considered significant if the *P* < 0.05. The symbol *was used to indicate a *P* <0.05, **indicated *P* < 0.01, ***indicated *P* < 0.001, and indicated *P* < 0.00001.

## Result

### Identification of strain

**Fig. 1:**
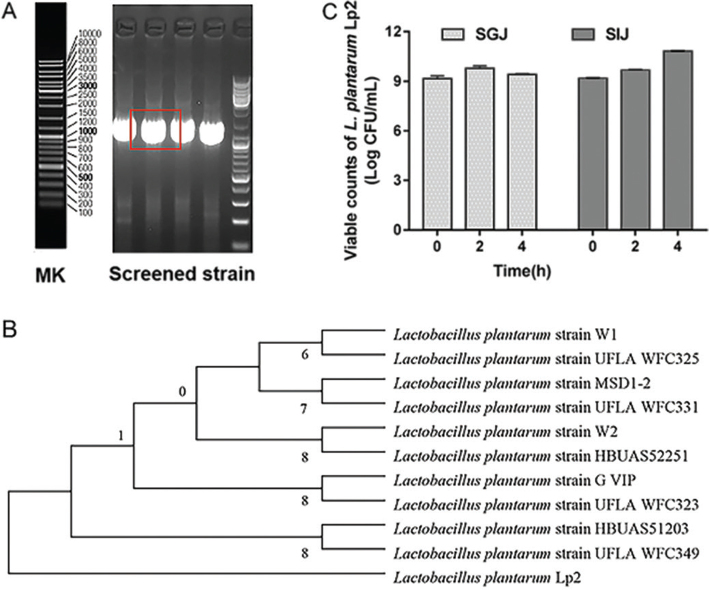
The identification of strains by the 16S rDNA sequence. The 16S rDNA sequence of *L. plantarum* Lp2. Electropherogram of screening strain (A), the phylogenetic Lp2 (B), and tolerance of screened strain Lp2 in simulated gastrointestinal fluids (C).

The strains were identified by colony morphological observation, cell morphology observation and 16S rDNA sequence analysis, which isolated from pickled cabbage. The images showed that the cells were Gram-positive, short rod-shaped, flagella free, and spore free. Homology comparison was performed in the GenBank database using Blast analysis. The 16S rDNA sequence of the pure strain is highly homologous to that of the *L. plantarum* strain, and their high identities are up to 99%, so it was identified as *L. plantarum* (Fig. 1B) and named Lp2 (CCTCC No. 2019935).

#### In vitro probiotic characterization of *L. plantarum* Lp2

*Antibacterial activity of L. plantarum Lp2. L. plantarum* Lp2 showed significant inhibitory activity against three indicator bacteria by agar well diffusion method (Table 1), including *E. coli*, *S. enteritidis* and *S. aureus* (zone of inhibition > 17 mm). Interestingly, for the control (pH 7.0), *L. plantarum* Lp2 still had an inhibitory effect on *S. enteritis* and *S. aureus*, which may be due to the bacteriostatic substances other than lactic acid was produced by *L. plantarum* LP2, such as bacteriocin (zone of inhibition > 9 mm).

**Table 1:** The antibacterial activity of metabolites in screening strains Lp2

Strain	Antibacterial circle diameter (mm)		
	*E. coil*	*S. enteritidis*	*S. aureus*
*L. plantarum* Lp2	22.90 ± 0.96	30.07 ± 5.14	17.43 ± 2.60
*L. plantarum* Lp2 (7.0)	0.00	18.81 ± 2.10	9.54 ± 0.85

Adjust *L. plantarum* Lp2 supernatant to pH = 7.0 as a control group

*Viability of L. plantarum Lp2 during exposure to simulated gastrointestinal juice.* In SGJ (pH 2.0), survival rates began to decrease about 0.3 log CFU, but there was no significant difference (Fig. 1C). In SIJ (pH 7.5), a tendency to retain viability was observed for all bacterial samples. Surprisingly, survival rates increased about 0.6 log CFU for 8 h.

Adhesion of *L. plantarum* Lp2 to Caco-2 cells. The adhesion capacities of the *L. plantarum* Lp2 strain were evaluated on Caco-2 cell monolayers. *L. plantarum* LSC3 and *L. plantarum* WCFS1 were used as a positive control, as it has good adherence compared with other commercial *Lactobacilli* (22, 23). The adherence was expressed as percentage, and values of 7.4 ± 1.5 and 11.2 ± 0.5% were observed for *L. plantarum* LSC3 and *L. plantarum* WCFS1, respectively. However, *L. plantarum* Lp2 showed a better adhesion effect, and the adherence was 36.0% ± 1.0%.

### Effects of *L. plantarum* Lp2 on organ index and LPS-induced lethality in mice

In order to study the immune and anti-inflammatory effects of *L. plantarum* Lp2, the liver injury model in LPS-induced mice was established. The survival rate of the mice in the LPS treatment group was 70%, and the 6-h survival rate of the Lp2 group was 100.0%. The mice in the LPS group showed signs of inflammation, such as lax fur, dehydration, diarrhea and lethargy, but these symptoms were not observed in the group treated with *L. plantarum* Lp2.

**Fig. 2:**
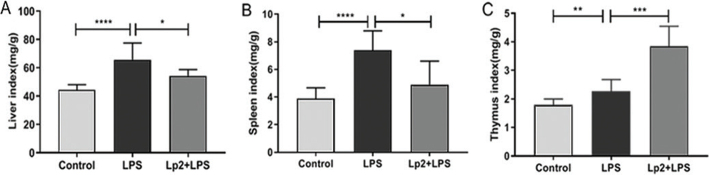
Effect of *L. plantarum* Lp2 on the organ coefficient of LPS-induced liver injury mice. Groups: control group, LPS group: intraperitoneal injection LPS after received diet for the liver index (A), the thymus index (B), and the spleen index (C) of mouse in the three r 4 weeks, and Lp2 group (Lp2 + LPS group): in addition to i.p. injection of LPS after 4 weeks, *L. plantarum* Lp2 was replenished daily with 1 mL. The mice in the LPS group presented signs of acute inflammation, such as ruffled fur, diarrhea, and lethargy, but these symptoms were not observed in the groups treated with Lp2. Values are represented as mean ± SEM (*n* =10). *P < 0.05, **P < 0.01, ***P < 0.001, and ****P < 0.0001 indicate the significant differences between different groups.

The indicators of LPS-induced mouse liver, thymus and spleen were evaluated (Fig. 2). Compared with the mice in the control group, the liver, thymus, and spleen indexes of the mice treated with LPS were increased significantly (*P* < 0.01). Oral *L. plantarum* Lp2 decreased the index of liver and spleen and increased significantly the index of thymus (*P* < 0.05).

### Effects of *L. plantarum* Lp2 on serum ALT and AST

**Fig. 3:**
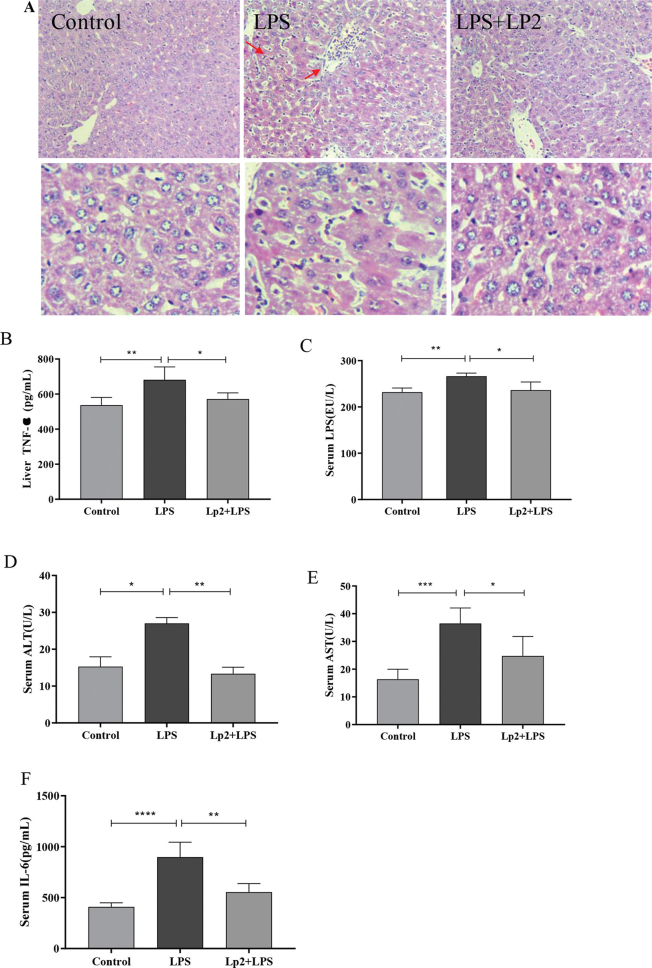
Pretreatment with *L. plantarum* Lp2 protected against LPS-induced liver injury. The histological changes of liver sections were measured by H&E staining at 200 × magnification (A). Control animals represent normal chow group; LPS-induced animals received normal diet for 4 weeks followed by single i. p. injection of 2.5 μg/kg LPS; Lp2 plus LPS-treated animals received diet with Lp2 for 4 weeks followed by single i. p. injection of LPS. Liver TNF-α (B), serum LPS (C), ALT (D), AST (E), and IL-6 (F) levels were measured by ELISA. Values are represented as mean ± SEM (*n* = 10). * *P* < 0.05, **P < 0.01, and ***P < 0.001 indicate the significant differences between different groups.

Compared with the control group, the levels of ALT (*P* < .05) and AST (*P* < 0.01) increased significantly in the LPS group (Fig. 3D–E). However, pretreatment with *L. plantarum* Lp2 once daily for 4 weeks reduced significantly the levels of ALT (*P* < 0.01) and AST (*P* < 0.05) compared with the LPS group.

### Effects of *L. plantarum* Lp2 on the pathologic symptoms of LPS-induced liver injury

Histologically, livers from mice injected with LPS showed extensive cell death in the H&E-stained sections (Fig. 3A). The results of H&E staining showed that liver tissues in the control group had uniformly arranged cells and clear nuclei. Compared with the control group, the LPS group showed more liver necrosis, hyperemia, and inflammatory cell infiltration, including slight inflammatory cell invasion in the portal area surrounding the blood vessels and a small amount of Kupffer cell proliferation and enlargement. The Lp2 group remarkably attenuated the appearance of cytoplasm damage and inflammatory cell infiltration.

### Effects of *L. plantarum* Lp2 reduced inflammatory factors in LPS-induced inflammation

In this study, to determine the effect of *L. plantarum* Lp2 on inflammation, expression levels of TNF-α, IL-6, and LPS in liver injury model mice were examined by ELISA analysis. As shown in Fig. 3, compared with the control group, the TNF-α, IL-6, and LPS levels in the LPS group were significantly higher (*P* < 0.01). Compared with the LPS group, the levels of TNF-α, IL-6 and LPS were significantly lower in the Lp2 group (*P* < 0.05).

### *L. plantarum* LP2 attenuated LPS-stimulated liver inflammatory responses via inhibiting MAPK phosphorylation

**Fig. 4:**
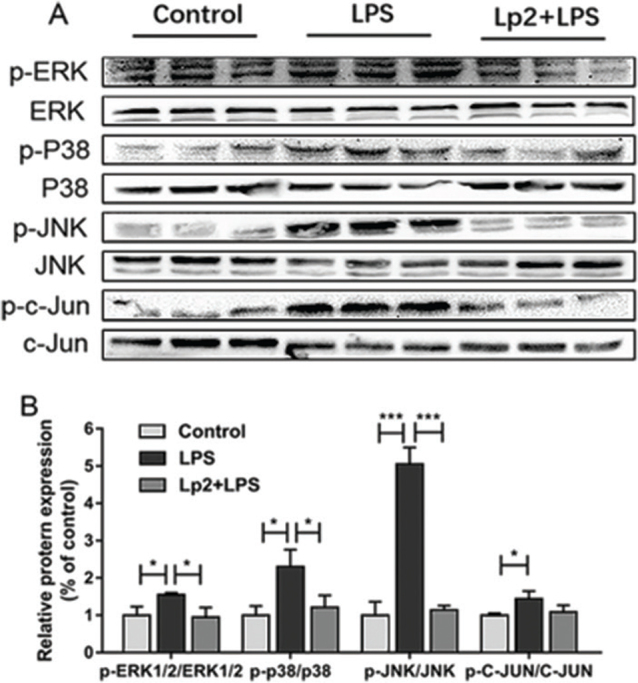
Effect of *L. plantarum* Lp2 on hepatic MAPK signaling. Livers of mice were collected 6 h after LPS challenge for Western blotting. p-ERK1/2, ERK1/2, p-p38, p38, p-JNK, JNK, p-c-Jun, and c-Jun protein expressions in liver. β-actin were used as a control for the protein blots (A). Quantification of ERK1/2, p38, JNK, and c-Jun phosphorylation (B). Values are expressed as mean ± SEM. *P < 0.05, ** *P* < 0.01, and ***P < 0.001 compared with the LPS group.

The MAPK signaling pathway plays a vital role in mediating LPS-induced inflammatory signal transduction and oxidative stress (24). The western blot analysis performed that the phosphorylation of p38, ERK1/2, JNK, and c-JUN increased in the LPS group compared with the control group (*P* < 0.05), whereas *L. plantarum* Lp2 inhibited significantly p38 ERK1/2, JNK, and c-JUN phosphorylation in the liver (*P* < 0.05) (Fig. 4).

### Effects of *L. plantarum* Lp2 on LPS-induced liver inflammatory response on TLR4/NF*κ*B pathway

**Fig. 5:**
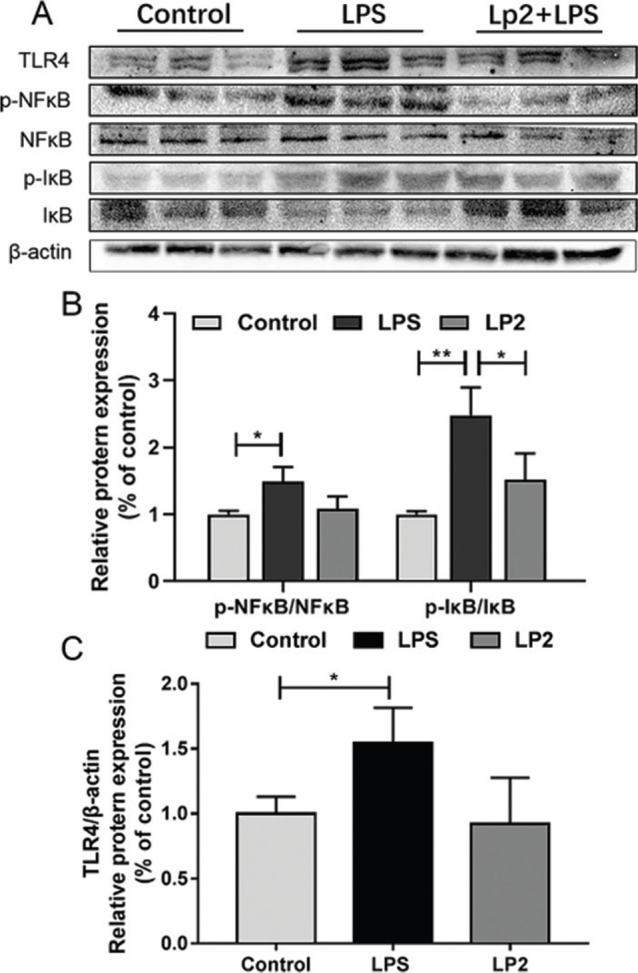
Effect of *L. plantarum* Lp2 on hepatic NFκB signaling. TLR4, p-NFκB, NFκB, I-κB, and Caspase-3 protein expressions in liver. β-actin was used as a control for the protein blots (A). Relative protein levels of TLR4(B). The levels of the phosphorylation of NFκB p65 were increased in the LPS group, Lp2 inhibited the LPS-induced increase of the phosphorylation of NFκB p65 (C). Values are expressed as mean ± SEM. *P < 0.05 and **P < 0.01 compared with the LPS group.

Western blotting was performed to explore the molecular mechanism of *L. plantarum* Lp2 on LPS induced, protein expression levels of markers associated with inflammation. TLR4, IκBα, and NFκB (p65) in the liver were determined (Fig. 5). Compared with the control group, treatment of LPS upregulated the expression of TLR4, increased the degradation of IκBα, and induced phosphorylation of NFκB (p65) (*P* < 0.05). However, the Lp2 group showed decreased expression of TLR4 and NFκB (p65) compared with that in the LPS group (*P* < 0.05). Furthermore, intervention with *L. plantarum* Lp2 reduced significantly the increase in Caspase-3 expression levels induced by LPS compared to the LPS group (*P* < 0.01) (Fig. 7).

**Fig. 6:**
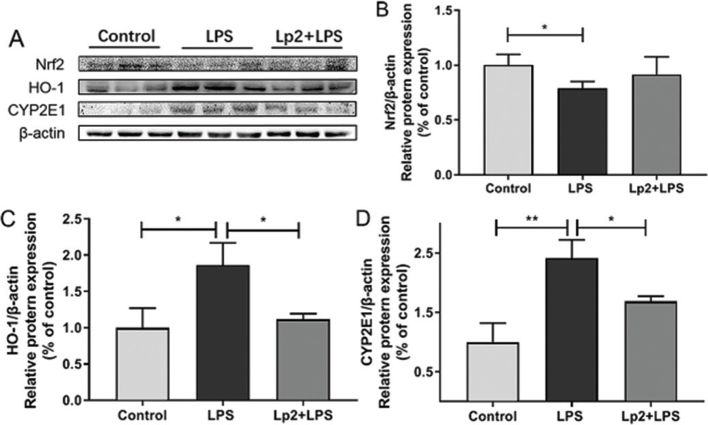
Effect of *L. plantarum* Lp2 on hepatic oxidative stress. β-actin was used as a control for the protein blots (A). Relative protein levels of Nrf2 (B). Relative protein levels of HO-1 (C). Relative protein levels of CYP2E1 (D). Values are expressed as mean ± SEM. *P < 0.05 and **P < 0.01 compared with the LPS group.

### Effects of *L. plantarum* Lp2 on the expression levels of oxidative stress-related markers in the liver

To further determine the anti-inflammatory mechanism of *L. plantarum* Lp2, we assessed the effect of *L. plantarum* Lp2 on oxidative stress in LPS-induced mice. The results showed that the expression of Nrf2 was downregulated significantly in the LPS group compared with the control group (*P* < 0.05). In comparison with the LPS group, the expression of Nrf2 increased significantly in the Lp2 group (Fig. 6A and B). Moreover, the similar result of HO-1 expression increased significantly after *L. plantarum* Lp2 treatment (*P* < 0.05) (Fig. 6A and C). CYP2E1 plays a critical role in LPS metabolism. Remarkable activation of CYP2E1 in the LPS group over than that in the control group (*P* < 0.05). The Lp2 group was found to significantly inhibit CYP2E1 activation compared with the LPS group (*P* < 0.01) (Fig. 6A and D).

**Fig. 7:**
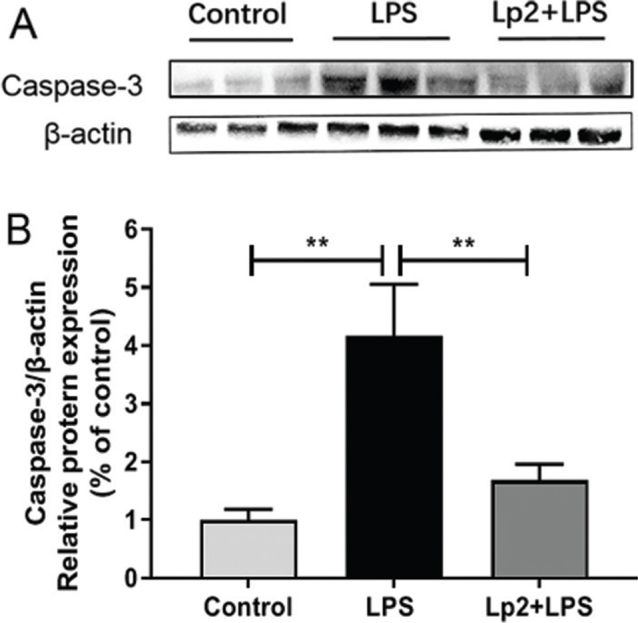
Effect of *L. plantarum* Lp2 on hepatic apoptosis. β-actin was used as a control for the protein blots (A). Relative protein levels of Caspase 3 (B). Values are expressed as mean ± SEM. *P < 0.05 and **P < 0.01 compared with the LPS group.

## Discussion

*L. plantarum*, as an important LAB, has received more and more attention in the fields of agriculture, food, and medical treatment. *L. plantarum* as a starter can increase the levels of folic acid, nicotinic acid, and vitamin B2 in food (25). Yogurt, cheese, sausage, and other agricultural products fermented by *L. plantarum* have improved greatly the nutrition and flavor of food (26). In addition to nutrition, *L. plantarum* can promote intestinal digestion, alleviate intestinal malabsorption, reduce serum cholesterol, anti-aging, anti-inflammation, and so on (27, 28). P. Kalac (29) showed that using *L. plantarum* as fermentation bacteria to produce kimchi can reduce the content of amine effectively in the product. A latest report showed that *L. plantarum* was able to induce antigen specific humoral, mucosal, and T--cell-mediated immune responses, providing efficient protection against coccidiosis challenge via oral vaccine delivery system (30). Moreover, Sefidgari-Abrasi S (31) studied that the mixed use of *L. plantarum* and inulin can be regarded as a novel adjunct therapy. However, the protective effects of the novel probiotic strain Lp2 against live injury have not been studied.

This study analyzed the viability of *L. plantarum* Lp2 in vitro by simulating the environment of the gastrointestinal fluid and found that its survival rate decreased very little in the extremely acidic gastric environment. Surprisingly, the survival rate of *L. plantarum* Lp2 did not continue to decline immediately in the intestinal environment, which may be due to its strong acid resistance and even cell recovery. Many studies indicated that certain probiotics can provide infection prevention capabilities for pathogenic microorganisms in the gastrointestinal tract (32–34). This study also found that *L. plantarum* Lp2 can inhibit Gram-negative bacteria *E. coli*, *S. aureus* and *S. enteritidis* effectively. Another desirable characteristic of probiotics is adhesion to human intestinal epithelial cells, as a close interaction between bacteria and host cells enables a transient colonization of the intestinal mucosa^27^. Thus, probiotics colonized in the intestine allowing antagonist effects against pathogens and host immune modulation (35). The screening strain Lp2, as a *L. plantarum*, has better adhesion than other commercial bacteria, and its Caco-2 cell adhesion rate is three to five times that of ordinary commercial bacteria (22).

In this study, an animal model of liver injury in mice induced by LPS was established. It was found that LPS induced would result in a significant change in body weight, liver, thymus, and spleen organs coefficient. The liver is one of the most common organs that respond to acute inflammation, and thymus and spleen are both the critically important immune organs to the human body. Xingyue Xu et al. (36) also showed similar effects that the coefficients of liver and immune organs increased in toxic mouse models due to the stress response. Compared with the LPS group, the treatment of *L. plantarum* Lp2 ameliorated acute inflammation by decreasing liver and spleen indices significantly. The increased thymus index should be a sign of an improved immune response by *L. plantarum* Lp2. Serum ALT and AST levels have been known as the key indicators for liver injury (37). In this study, *L. plantarum* Lp2 decreased the concentration of AST and ALT in plasma and alleviated the histological changes of liver.

Accumulated evidence has confirmed that LPS could activate Kupffer cells and induce the expressions of inflammatory cytokines by TLR4-mediated activation of the MAPK and NF-κB pathways. On the one hand, in resting cells, NFκB dimers remain inactive by association with inhibitory proteins of the IκB family. When signaling pathways were activated, the IκB protein was degraded, which was mediated by the IκB kinase (IKK) complex, which led to phosphorylating IκB, triggering their ubiquitination and proteasomal degradation. Then NFκB dimers entered the nucleus to modulate the expressions of genes associated with inflammation including TNF-α, IL-6, and IL-8 (38). On the other hand, the recognition of LPS by TLR4 induces phosphorylation of MAPK signaling cascades, including p38, ERK, and JNK, which plays an essential role in the expressions of inflammatory cytokines such as TNF-α and IL-6 (39). Our previous research showed that *L. plantarum* LP104 led to a significant reduction in liver AST, ALT, and TNF-α (3). TNF-α, together with other alarm proinflammatory cytokines IL-6 and IL-1β, is known to be required for the induction of inflammation (40, 41). Wang et al. (42) showed that the mediation of *Bifidobacterium longum* R0175 can reduce the expression of TNF-α, IL-6, and chemokines significantly in a rat model of LPS/D-GalN-induced acute liver failure. *L. plantarum* Lp2 downregulated the LPS-induced phosphorylation of IκB-α, resulting in the proteasomal degradation of IκB-α, and suppressed NFκB (p65) phosphorylation; moreover, *L. plantarum* Lp2 also decreased the expressions of TLR4 and MAPKs (c-Jun, ERK, JNK, and p-38 MAPK) in liver. Therefore, the decrease of inflammatory cytokines TNF-α and IL-6 in the serum of mice treated with *L. plantarum* Lp2 by inhibiting MAPK/NFκB signaling pathways. Similarly, previous studies have demonstrated that probiotic *L. casei* Zhang attenuated LPS/GalN-induced liver inflammation through inhibition of ERK, JNK, and p38 MAPK phosphorylation, induction of TLRs expression, and inflammatory mediators (i.e. TNF-α and IL-1β) (40).

Likewise, *L. plantarum* C88 downregulated the expression of NFκB in a LPS/GalN-induced acute liver injury mouse model, which decreased the levels of TNF-α, IL-6, and IL-12 in liver (43). Based on this, it was shown that *L. plantarum* Lp2 has potential relief and prevention effects on LPS-induced liver injury.

Specifically, cell apoptosis is an important factor in cell death (44); intestinal epithelial cell undergoes apoptosis as a result of LPS stimulating mice to trigger acute inflammation. It has been reported that the permeability of the epithelial barrier is related to apoptosis induced by Caspase-3 activation. In fact, Caspase-3 functions as a central effector (45). *L. reuteri* ZJ617 supplementation reduced apoptosis induced by LPS challenge (46). At the same time, it was showed in vitro that *L. plantarum* 299v prevented Caspase-dependent apoptosis (47). To our knowledge, there have been few reports of *L. plantarum* species regulating apoptosis in animal models of inflammation. In this study, *L. plantarum* Lp2 mediated the expression of Caspase-3. This is the first time to demonstrate the anti-apoptotic properties of *L. plantarum* Lp2 in LPS-induced mice clearly.

Oxidative stress and inflammation are interacting processes. It has been shown that oxidative stress causing apoptosis and inflammation was attributed to the interaction of multiple pathways (48). Several studies have reported that downregulation of HO-1 expression severely exacerbates liver injury caused by LPS (9, 49). Nrf2 upregulates HO-1 by binding to antioxidant response elements, inducing cytoprotective adaptive responses and having an important effect on the response to oxidative stress (50). In this study, *L. plantarum* Lp2 increased significantly the nuclear level of Nrf2 and further upregulated HO-1 expression. Noticeably, excessive CYP2E1 expression against LPS-mediated animal models in this study is consistent with others (4). If ROS is produced in excess, oxidative stress will increase and cause damage to cells through direct or indirect pathways. In this study, *L. plantarum* Lp2 showed strong inhibition on LPS-induced overexpression of CYP2E1 by Western blotting analyses. Taken together, these results suggested an antioxidant effect of *L. plantarum* Lp2 on the LPS-induced liver injury.

In summary, *L. plantarum* Lp2 isolated from pickled cabbage possessed probiotic properties including the tolerance of simulated gastrointestinal tract, effective adherence abilities, and anti-pathogen activities. And *L. plantarum* Lp2 exerted anti-inflammatory effects by inhibiting the inflammatory cytokine production and reducing the activity of the TLR4 and MAPK pathways, resulting in the inhibition of NFκB, particularly in association with the activation of Nrf2/HO-1 signaling. The results revealed that *L. plantarum* Lp2 improved LPS-induced liver injury by ameliorating antioxidant, inflammation responses, and apoptosis.
